# St. Louis Medical Society

**Published:** 1880-05

**Authors:** 


### ST. LOUIS MEDICAL SOCIETY.

*On the Relations of the Placenta to Post-partum Hemorrhage.*—Dr. Walter Coles read a paper to the Society making these points: "As a rule it will be found that the profuseness of the hemorrhage will be in direct ratio to the uterine inertia, and inversely proportional to the area of adherence of the placenta; that is to say, the flow of blood will be in direct proportion to the laxity of uterine fibre, and to the extent and number of ruptured vessels in the artero-placental site. If this be true it is evident that in ordinary labor the presence of the placenta *in*

*utero* can only promote hemorrhage, in so far as it offers a mechanical obstacle to uterine contraction, whilst it really lessens it to the extent of its *adherent surface.* Although we are aware that this latter proposition is in direct conflict with high authority, we think it tenable on physiological and clinical grounds, and hence we are justified in concluding that the only possible good which can accrue from the speedy delivery of the after-birth in *post partum hemorrhage* is the new mechanical one of the cleaning of the cavity of the womb of so much foreign material in the same manner, and for the same reason, that we would sweep out so much clotted blood, simply to get rid of it,and in order to clear the way for local styptics within the uterine cavity."

"Entirely too much stress is being laid upon the retention of the after-birth, and too little upon the fundamental fact that the placenta has in nine cases out of ten nothing whatever to do with the bleeding, which is rather due to absence of uterine contraction—to *inertia.*"

Dr. Coles then presents the following propositions:

1. The placenta can only participate in causing post-partum hemorrhage when it is either of abnormal size, or abnormally adherent, thus offering a physical bar to uterine cantraction.

2. Simple retention of the platenta, when detached or partially adherent by *normal* attachment, does not of itself contribute to the production of hemorrhage.

"To my mind," continued Dr. Coles, "abnormal adhesion, or abnormal size of the placenta, constitutes the only valid reason for its manual detachment and delivery as a curative means in post-partum hemorrhage, in as much as these cover the only conditions in which the placenta is a factor in the production of bleeding."

*Discussion.*—Dr. W. Johnson said he could not accede to the second proposition, that the placenta partially detached was not actively concerned in hemorrhage. The placenta was partially connected with the uterus, and the circulation was going on ; it must produce hemorrhage. In that condition the accoucher should use all means possible to bring about uterine contraction. He might introduce his hand, and stop the flowing at once. He could not be justified in that course of action unless he was satisfied it would produce uterine contraction. The next best course was to stimulate the nerves running through the uterus; ergot was useful; cold application would bring about contraction.

Dr. G. J. Englemann:—I am glad to hear that paper. I think, as a rule, post-partum hemorrhage and retention of placenta are consequences of uterine inertia. It is true most text books say, "Remove the placenta," and some writers not in text books insist upon the instantaneous removal of the placenta in post-partum hemorrhage, provided the hemorrhage occurs before the removal of the placenta. It is generally true that after the removal of the placenta, the hemorrhage ceases. But why? Either the placenta is removed by contractions of the womb, excited internally, or the hand is introduced into the womb, and it excites uterine contraction. If we apply pressure externally, we may excite contraction; we may introduce one hand, and with it compress the uterine fundus. That is an excellent way of stimulating contraction. Our object is to stimulate uterine contraction and the placenta is removed. I suppose there are cases where the retention of the placenta will cause hemorrhage, possibly where there is adherent placenta, and yet if the uterus contracts well, there will be no serious hemorrhage.

Dr. W. L. Barret.—These are innovations of Dr. Coles; the idea of having the placenta in the uterus with impunity for an indefinite time, or the idea of not removing the placenta, for the preventing of hemorrhage or the checking of hemorrhage. I wish there was more time to discuss the subject. I think it deserves to be fully discussed. If the uterine contraction is at all firm, the vessels are sufficiently ligated to stop the flow of blood. Anything that prevents the contraction of the uterus, will increase the chances of hemorrhage, and so long as the placenta or any mass remains there, preventing the contraction, the danger of hemorrhage will be increased. It does not matter whether it is a tumor or something adherent to the organ; it increases the danger of hemorrhage. I do not pretend to dispute that there are a great many cases in which the placenta is probably partly separated from the uterus, and yet there is no excessive hemorrhage. The placenta is almost always in part separated, as in the last contractions, that expel the child.

Dr. Atwood.—I am satisfied that in all cases of post-partum hemorrhage there is a failure on the part of the uterus to contract. During the discussion I have recalled certain cases that fully convince me of the correctness of Dr. Barrett's position. You must have firm contraction to close the mouths of the blood vessels. I remember one case in which, after delivery, following the doctrine I had then read, to immediately remove the placenta, I carried my hand up, found the placenta within the os, put my fingers around it and removed it, supposing I had removed the entire placenta. I did not, as I think every obstetrician should, examine to see if any portion was left. My patient was attacked with an alarming hemorrhage. I immediately made firm compression on the uterus and applied cold water. I gave her brandy and water, but all to no purpose. It occurred to me I might have left a portion of the placenta in the uterus, introduced my hand, removed the remainder of the placenta, only about two inches, and the trouble ceased immediately. My patient made a good recovery. I wish to cite another case that has often caused me thought and trouble. I was engaged to deliver a certain woman, and being out of town, a neighboring midwife was called in. I visited the house and asked if the afterbirth had been removed; was told it had been. I gave the usual directions for management and left. I heard nothing more of the case for seventeen days, and was then sent for. I found her flooding violently. I gave her ergot and stimulants, but to no purpose. Then I introduced my hand and found a tumor near the fundus. With some difficulty I detached a large saucerful of what appeared to be the placenta. The hemorrhage immediately ceased; there was no further trouble. These are proofs posit ve to me that the uterus could not contract as long as there was a portion of the afterbirth unremoved.

Dr. Coles.—I am sorry that I have read this paper so near the hour of adjournment. [He read extracts from his manuscripts and asserted that he did not advocate the retention of the placenta for an indefinite time; he said it need not be delivered as a necessarily curative method in flooding.]

The further discussion of the subject was postponed.

*Removal of the Placenta.* Dr. Coles.—I trust the Society will permit me to refer to the subject under discussion at the time we adjourned last Saturday night. I read a paper at a late hour, and some points made in my paper I think, perhaps, were not clearly understood by some gentlemen. I am sorry, indeed, if any member of the Society should think I advocate absolute non-interference with the placenta in all cases of post-partum hemorrhage, especially as regards its relation to the placenta. My object was to point out the true causes of post-partum flooding, and to draw attention to the fact that in many of the most dangerous of these cases the placenta is not an *active agent* in its production, and hence, that it is important before proceeding to deliver the afterbirth to determine whether the fault lies with it, or whether it is not due to uterine inertia. In which case, I contended, that harm rather than good might result from premature detachment and delivery of the placenta from a flabby, unconscious uterus.

*The use of pessaries.*—D. William Johnson drew a diagram on the blackboard illustrating the relative positions of the uterus, rectum and bladder. He said the subject upon which he desired to speak was an old one, on which he differed from many of the higher authorities. He expressed his belief that the pessary did harm rather than good; and called attention to the comparative distances between the arch of the pubes and the sacrum, in front of which was the rectum, arguing that if the pessary was used as a fulcrum, it could not, according to the law of physics, do the good that was claimed for it. In his opinion the only pessary that could do good was the *stem* instrument of Simpson, introduced through the cervix into the body of the womb; that would hold the womb in position. No ring pessary could avail anything; he had abandoned them entirely. When there was relaxation of the vaginal walls, he used carbolic acid and sulphate of zinc, which tended to contract its tissues, and thereby give some support to the body of the uterus.

President Maughs said that Dr. Johnson had opened an interesting subject, upon which he was not quite alone in his views, since some respectable authorities held similar tents.

Dr. Johnson said that he did claim that the diagram was scientifically drawn.

Dr. G. Hurt: I was going to take exception to the diagram upon the ground that it is erroneous, and does not represent the normal position of the part, which seem to be out of the true line. Instead of the uterus being a little posterior to the vertical line as represented in that diagram, it is just the reverse. And while it is true the application of the pessary sometimes increases the risk of flxion or misplacement of the uterus, producing that which it is used to remedy, yet I am not disposed to reject that instrument entirely. Upon all forms and under all circumstances, I have used the pessary very moderately in my professional career, and, I have to say in some cases, I was satisfied it was not useful, but I have introduced it in other cases in which the patient seemed to express satisfaction. I treated a lady some few years ago, who was subject to very painful and excruciating paroxysms of dysmenorrhœa, and, with other remedies used in that case, I applied a pessary. As far as examination revealed, the trouble arose from a retroflexed and tortuous cervix. This lady entirely recovered, and has since borne children.

Dr. Fairbrother: Do I understand Dr. Johnson to say that is is the stem pessary which is the greatest humbug ever foisted on mankind?

Dr. Johnson: No, sir; the only one that can do any good is Simpson's stem pessary; and it should be watched with a good deal of care. All the others I condemn.

Dr. Fairbrother: Mr. President, this is the same line of argument that was followed in a paper which was read here last fall. In this paper, and I believe in some of the remarks which followed it, pessaries were denounced as a dangerous imposition upon credulous women, and as mere toys to tickle the fancy.

It was certainly with a feeling, something akin to pain, that I listened to this sort of talk, because at that time I had under my care a remarkable illustration of the good effects following the use of the pessary. It was in the case of a woman who made her living by washing. The uterus was prolapsed so that the os projected beyond the external labia. The condition had been growing for years, until at last she was disabled from work and sought relief. The uterus was replaced, a simple ring pessary being introduced into the vagina, and the woman returned to her work entirely free from the pains and forebodings that were rapidly wearing out her life. This is the only case; you have all seen similar ones. I am speaking only of procidentia—saying nothing of the vast of good to be obtained by their skillful use in the various flexions and versions. There certainly can be no doubt of their utility in such a case as the one I have instanced.

Dr. Coles: I think there is no doubt but that the pessaries are often employed when they are really of no advantage, and sometimes when they are a positive disadvantage to the patient; but there are many cases, as remarked by Dr. Fairbrother, when a well-fitted pessary is a *sine qua non.* I am remined of a case, where during the past week, I have enabled a woman to resume her daily duties whose life was before that a burden to her on account of prolapsus. It must be judiciously selected and properly applied. The same instrument does not suit every case, nor does every case of womb trouble demand a support of this kind. Dr. Johnson is in error both in his premises and conclusions; his drawing incorrectly represents the relations of the parts, and he is entirely at fault as to the manner in which the ordinary pessary subserves its purpose. With such crude views of their application, it is not remarkable that he has become discouraged with their use. The doctor represents the ordinary lever instrument of Smith and Hodge as resting upon the pubes anterioirly and passing upwards and backwards in a straight line to the hollow of the sacrum, the upper and posterior extremity pressing directly against the rectum. This, however, is not as it should be. The pessary, like the forceps, must be adapted to the curves of the vagina and pelvis, as without these, and without perfect adjustment, either instrument is capable of harm.

The normal anatomy of the pelvic organs must be kept clearly in mind, as well as the mechanism of uterine displacements, before any attempt is made at reposition and retention of the womb by mechanical means. I prefer the pessary of Hodge or Smith to any others. In both of these instruments there is a double curve, well adapted to the natural curve of the vagina. This curve is essential, not only to keep the instrument in place, but to the comfort of the patient; resting against the pubes in front, it projects backwards and upwards, not directly against the rectum, as Dr. Johnson aserts, but curves upward until its lateral bars lie parallel with this organ, while the superior cross-bar of the pessary rests in and supports the vaginal fold reflected between the rectum and uterus posteriorily. Thus adjusted, and if of proper length, the posterior pressure of the instrument, instead of being confined to one point, is shared partly by the rectum, but chiefly by the superior-posterior *cul-de-sac* of the vagina. The instrument, thus placcd, has no absolute fixed point of rest posteriorly, but slides gently up and down with every motion and respiration of the patient. Great judgment and discrimination should be exercised in selecting pessaries; sometimes a number have to be tried before a proper fit is secured. To act comfortably and physiologicaily it must have the proper curve; if too short it is worthless; if too long it is painful and may become dangerous.

My experience with the stem pessary has not been large, but from what I know of it, when I have occasionally employed it for amenorrhœa, I would unhesitatingly condemn the instrument as impracticable and hurtful. As to anteversion pessaries, I have never seen one yet that would accomplish and secure the desired end. It should be remembered, however, that no pessary is intended to correct a malposition of the uterus by its own inherent power. Its province is to assist in holding the uterus in its proper place, after the physician has reposited it. This step should always precede the introduction of the pessary, and should be thoroughly done, a point which is frequently overlooked both in debate and in practice.

Dr. T. F. Prewitt: I think Dr. Johnson's condemnation of pessaries in general is too sweeping, and his indorsement of the stem pessary too generous. I agree with him in the fact that in many cases we find great difficulty in supporting the uterus with the pessary proper. If you take a retroflexed and retroverted uterus, I think all of us will agree that the pessary answers our purpose very indifferently in carrying it up and forward. By reason of this very insufficiency in the leverage we bring to bear upon it, the fundus under these circumstances is probably thickened, congested and the pessary does not reach high enough to lift up the weight of the body of the uterus and carry it forward. But in a simply retroverted uterus that is disposed to tip back when one places it in position, I think in a majority of cases a well adjusted pessary would retain it there. Where we have a retroflexed uterus, Dr. Johnson would dispense with the pessary, but he does not tell us what he would do. As to the stem pessary, I think it a dangerous instrument.

Dr. Bond said that he had no doubt but that pessaries served a good purpose. He was satisfied that pessaries had their greatest application in retroversion rather than in retroflexion. The instrument must be adapted to the curvature of the vagina. It must be such that when the force is exerted upward it may be between the uterus and rectum, neither too much against the one nor the other. The instrument is kept in place and assisted in its work as a supporter by the natural contractility of the vagina, which is normally a closed canal, its walls possessing decided muscular contractility, and these muscular fibres so arranged that their tendency is to contract from below upwards. thus exerting an upward pressure upon the pessary which is transmitted to the uterus.

Dr. C. W. Stevens condemed the use of pessaries when they had been employed for the purpose of correcting any other malposition of the uterus than prolapsus. He did not believe that the mere introduction of any of the Hodge modifications of pessary could correct even temporarily any malposition; that it might for a short time hold the uterus in a certain position, he could not dispute, but that it corrected a malposition he very much doubted. The fulcrum or lever rested on an unstable foundation; the rectum was full at one time and empty at another. He conceded that in some cases the ring pessary had afforded great relief, but failed to recognize the utility of Hodge's instrument.—*St. Louis Medical and Surgical Journal.*

